# Supplementary material for: Are carbon emissions trading and green financial instruments synergistic? -Comprehensive quantitative research based on content analysis
Source: PLoS One. 2024 Mar 7;19(3):e0298601. doi: 10.1371/journal.pone.0298601 (PMC10919601; doi:10.1371/journal.pone.0298601)
Supplement: S1 Data — (ZIP) [file pone.0298601.s001.zip › Data/地级市Z_F工作报告.zip!/╜¡╦╒/┴1⁄4╘╞╕█╩╨/└┤╫╘╣1⁄2╓┌║┼í╛┬φ┐╦╩2╛▌═°í┐_2016.docx]

各位代表：

现在，我代表市人民政府向大会作工作报告，请予审议，并请各位政协委员提出意见。

十二五时期工作回顾

五年来，全市人民在省委、省政府和市委的正确领导下，凝心聚力，攻坚克难，开拓进取，推动经济社会发展取得长足进步。全市战略地位不断提升，综合实力日益增强，产业发展、城乡面貌、港口建设、人民生活发生了显著变化。

五年来，我们坚持狠抓第一要务，加快推动产业发展，经济建设迈上新台阶

综合经济实力明显提升。全市GDP突破2100亿元，预计年均增长11.6%；固定资产投资突破2000亿元，年均增长20.8%；一般公共预算收入达到291.8亿元，年均增长16.4%；社会消费品零售总额达到830.7亿元；城乡居民收入年均分别增长10.3%、12.8%。

三次产业持续快速发展。现代农业发展成效明显，粮食生产实现十二连丰，高效设施农业面积翻番，形成食用菌、泥鳅等十大特色主导产业，创成花果山农业出口示范区、赣榆现代农业示范区等国家级农业园区4家，省级以上龙头企业达到50家，获批国家地理标志保护产品9个，数量位居全省前列。工业经济扩量提质，规模以上工业增加值达到1157亿元，是十一五末的2.5倍。新医药、新材料、新能源产业产值达到1500亿元，装备制造、石化产业产值突破千亿规模。现代服务业快速发展，物流产业产值年均增长26.1%，文化产业增加值年均增幅超过30%，房地产市场平稳发展。新增国家4A级景区7家，花果山景区获批全国旅游标准化示范单位，温泉度假区创成省级旅游度假区。商贸服务业提档升级，大润发、苏宁广场等一批大型商业设施建成投用。

重大产业项目取得突破。净增规模以上工业企业470家。虹港石化、亚新钢铁等项目建成投产并跻身全市工业产值前20强。产值超百亿元企业达7家，总数处于苏北前列，其中新海石化、镔鑫特钢产值突破200亿元。石化产业基地和炼化一体化项目获得国家批准开展前期工作。田湾核电站3、4号机组进入设备安装阶段，5号机组开工建设。

五年来，我们深入推进沿海开发，积极融入一带一路建设，战略地位不断提升

重大基础设施得到完善。组合大港基本形成，建成30万吨级深水航道一期工程，赣榆、灌河、徐圩两翼港区开港运营，综合通过能力达到1.6亿吨，为十一五末的1.5倍。新改建国省干线公路近500公里，总里程实现翻番，连盐、连淮扬镇、连青铁路开工建设，徐连高铁列入中铁总公司年度开工计划，疏港航道、盐河航道建成通航，实现千吨级船舶直通京杭大运河。新机场建设取得重要进展。电网建设完成固定资产投资93亿元，较十一五增长72.2%。这些为港城发展提供了有力保障。

综合口岸功能得到提升。中韩陆海联运列为全国试点。赣榆、徐圩港区实现临时开放，白塔埠机场获批一类开放口岸。与新加坡国际港务、巴西淡水河谷开展全面战略合作，新开通加密38条近远洋航线。海铁联运开通至霍尔果斯、西宁等国内班列，连新亚、连新欧班列开通运营。在淮安、洛阳等地设立无水港9个。港口吞吐量、集装箱运量分别达2.1亿吨、501万标箱，承担了全国50%、中亚国家60%以上的大陆桥过境运输业务，海铁联运成为连云港的重要国际品牌。

开放载体建设取得突破。抢抓机遇、主动对接，被国家确定为新亚欧大陆桥经济走廊首要节点城市、中哈物流合作基地和上合组织出海基地。在两国元首共同见证下，中哈物流合作基地项目一期建成启用。上合组织国际物流园建设全面推进。国家级、省级开发园区转型发展成效明显，成为全市经济发展的主阵地。国家东中西区域合作示范区获批，先导区建设全面拉开，一批开放合作平台载体建成投用。成功举办两届连博会。

开放型经济水平不断提升。累计利用外资40亿美元，年均增长近17%。外贸进出口377亿美元，较十一五实现翻番。高新技术产品出口以年均15%速度递增，农产品出口跃居全省首位。新批境外投资项目71个，协议对外投资额达12.6亿美元，企业走出去步伐明显加快。

五年来，我们着力深化重点领域改革，大力实施创新驱动战略，内生发展动力不断增强

重点改革纵深推进。推进政府机构改革，组建卫计委、外侨办，完成工商、质监、食品药品管理体制调整。深化行政审批制度改革，行政许可项目削减至97项，行政审批项目集中办理率达100%。启动三证合一登记制度。全面推进政务公开，行政审批事项目录清单、政府行政权力清单等五张清单上网公布。加快国有企业改革，整合组建六大集团。启动三互三个一口岸大通关体制改革，实现船舶进出港数据一单五报。深化农村改革，东海农村产权交易平台、县乡村三级土地流转服务网络、农村信用体系建设等多项改革经验全国推广。深化财税、价格改革，初步建立政府全口径预算制度，政府性债务管理进一步规范，市场决定价格机制进一步健全。深化金融改革试点，5家地方法人金融机构完成商业银行改制，新增上市公司3家，新引进银行业金融机构8家，4家企业获得外汇资金池和跨境人民币资金池集中运营资格。深化医药卫生体制改革，公立医院全部实施药品零差率销售，国家基本药物制度全面实施。

创新驱动战略深入实施。先后被确定为国家创新型试点城市、国家知识产权试点城市，连续获评全国科技进步先进市。全社会研发投入占GDP比重由1.17%提高到1.65%，科技进步贡献率由41%提高到53%，高新技术产业产值年均增长26%。创新能力显著增强，重大新药创制、高强高模碳纤维、IGCC等一批关键核心技术实现突破，荣获国家科技进步一等奖1项、二等奖6项、中国专利金奖3项。恒瑞医药成功向美国市场转让创新药品，康缘药业智能制造经验全国推广，正大天晴、豪森药业等4家企业跻身全国医药企业创新力20强。创新载体建设成效明显，连云港高新区升格为国家级高新区，获批建设硅材料等4个国家级特色产业基地，建成国家级企业研发机构4家、质检中心1个。新增国家级博士后科研工作站5家，引进省双创团队4个、双创人才51人。

五年来，我们不断优化城市功能布局，全面推进城乡一体化建设，海港城市特色日益彰显

城市布局日趋优化。明确港产城融合发展思路，科学界定各城区、各功能组团定位。顺利完成新浦、海州两区合并，赣榆撤县建区。连云区与连云新城、徐圩新区与板桥工业园实现一体化发展。120公里的海滨大道全线贯通，形成串联沿海的快速连接线，山海相拥、港岛辉映的海滨特色得到彰显。建成全长34公里的城市快速公交1号线，有效拉近了东西城区的时空距离。建设环云台山大道42公里，开启了揽山入城新篇章。徐新公路全线贯通，310国道市区段建成通车，204国道绕城段路基全面建成，市域道路网络日趋完善，交通出行更加便捷。市区建成区面积扩大到204平方公里。

城市建设成效显著。建成市体育中心、科创城一期、工业展览中心等重大服务设施，完成民主路、盐河巷、连云港老街等特色街区改造。完善市政公用配套设施，新建和改造供水、污水、燃气管网300公里。实施城区道路提标改造工程，新建改建主次干道51条，打通城市断头路　15条。新增市区公交线路30条，公交出行分担率提高到23%。投资27亿元实施城市饮用水双源双线双湖工程，沭新渠送清水工程建成投用，蔷薇湖应急备用水源主体工程建成。完成棚户区改造380万平方米，改造老旧小区135个。启动实施城市防洪排涝提升工程，整治低洼易涝片区28个、背街小巷1800条、170公里，较好地解决了汛期积水问题。新建月牙岛、星海湖等公园12座，中心城区绿地总量增加到6292公顷，比十一五末增长60%。刘湾垃圾填埋场一期工程建成使用，新建改建公厕150座。建筑工地及道路扬尘管控经验全省推广。

新型城镇化加快推进。全市城镇化率达61%，较十一五末提高14个百分点。各县城区规模、功能、形象显著提升，综合承载力日益增强，人口均突破20万。重点中心镇产业和人口加速集聚，13个城镇列入省级以上培育计划。开展美丽乡村建设行动，创成三星级康居乡村66个，建成美丽乡村试点38个。推进城乡基础设施和公共服务一体化，开展通榆河、新沭河等流域性河道整治，实施新一轮海堤达标工程，完成在册大中小型水库和市区病险塘坝除险加固，疏浚县乡河道424条。行政村客运班车通达实现全覆盖，镇村公交开通率达48%。新建城镇污水处理厂36座、垃圾转运站88座。

生态建设持续加强。完成国家淮河流域水污染防治规划项目20个，实施城市河道环境综合整治8条，城乡水环境质量明显改善。完成省定黄标车和老旧机动车、燃煤锅炉、挥发性有机物整治任务，实施秸秆禁烧网格化管控，空气质量优良率全省领先。开展沿海化工园区环保专项整治，关闭、停产、限期治理一批化工企业。秦山岛、连岛整治修复取得成效，海州湾获批国家级海洋公园。实施绿化港城行动，新造林106万亩，林木覆盖率提高4.5个百分点，获评国家园林城市。在全国地级市率先开展战略环评试点，东海县通过国家级生态县考核验收，赣榆区通过国家级生态区技术评估。花果山景区获批省级生态旅游示范区。

五年来，我们坚持推进民生改善，加强社会管理创新，人民生活水平不断提升

人民生活持续改善。健全完善就业创业政策体系，新增城镇就业人员41.3万人，农村劳动力转移就业20万人，高校毕业生就业率稳定在90%以上，城镇零就业、农村零转移家庭保持动态为零，期末城镇登记失业率为2.1%。扶贫攻坚成效明显，重点推进石梁河库区、新沂河两岸片区扶贫开发，实施帮扶项目526个，总投资达34亿元。46.1万农村低收入人口、254个经济薄弱村整体实现脱贫，本轮脱贫奔小康工程圆满收官。城镇居民人均可支配收入达到25800元，农民人均收入达到12900元。

社会保障得到加强。城镇职工五险参保人数较十一五末增长35.9%，城镇医疗保险、新型农村合作医疗实现应保尽保，城乡低保标准分别提高81%、130%。全面落实困难群体救助扶助政策，累计发放各类资金38.4亿元。住房保障体系逐步完善，开工建设各类保障性住房4.18万套，发放廉租房补贴8219户，惠及5万多户中低收入家庭。累计发放住房公积金贷款122亿元。公办示范性养老机构实现全覆盖，每千名老人拥有床位数达32张，赣榆农村社区集中养老模式全国推广。

社会事业协调发展。累计投入资金100多亿元，建成连云港实验学校、公共卫生服务中心、民政公共服务中心等一批重点基础设施。全部建成义务教育发展基本均衡县（区），在苏北率先基本实现教育现代化。成功引进康达学院，财经学校实现升格，连云港师专开展本科联合办学。市直三级医院实现全覆盖，社区卫生机构实现以街道为单位全覆盖，新增二级专科医院13家。县级人民医院、中医院实现二级甲等全覆盖，85%乡镇卫生院达到省级标准。建成城市十分钟健身圈，人均拥有公共体育场地面积从1.87平方米增加到2.75平方米。率先完成省级公共文化服务标准化试点地区创建，科技馆实现县区全覆盖，万人拥有公共文化设施面积从十一五末的660平方米增加到1200平方米。组织文体惠民活动3万余场，举办西游记文化节、第八届中国曲艺节、徐圩国际马拉松等大型活动40余项。

社会治理扎实推进。大力弘扬社会主义核心价值观，涌现出雷锋车组、开山岛夫妻哨、最美基层干部姜霜菊等一批全国先进典型。坚持依法行政，政府效率效能得到提升。加强基层基础建设，政社互动覆盖75%的乡镇，一委三会社会治理模式在全省推广。实施六五普法规划，建成省级以上民主法治示范村（社区）543个。建立健全社会治安防控体系，严厉打击刑事犯罪，群众安全感持续增强。创新矛盾纠纷多元融合调处机制，推进信访工作制度化、规范化、法制化建设，有力促进了社会稳定。质量强市全面推进，食品药品安全稳中向好，安全生产形势总体平稳。推进军民融合发展，实现全国双拥模范城七连冠。政府服务热线受理处置各类问题100余万件，群众满意度达90%以上。民族宗教、档案史志、妇女儿童、残疾人、红十字、关心下一代、老龄事业等取得新进步。

刚刚过去的2015年，面对经济下行压力不断加大的严峻形势，我们主动适应经济发展新常态，扎实做好稳增长、调结构、促改革、惠民生各项工作，全市经济社会发展取得了新的成绩。预计2015年全市地区生产总值增长10.8%，其中规模以上工业增加值增长12.9%，服务业增加值增长11.5%。固定资产投资增长21%，其中工业投资增长21.7%。一般公共预算收入增长11.5%，社会消费品零售总额增长12.4%。城乡居民收入分别增长9.5%、10%。民生百件实事顺利实施。总体看，基本完成了市十三届人大四次会议确定的各项目标任务，主要指标好于去年同期、高于周边城市，规模以上工业增加值、外向型经济、城乡居民收入等指标虽低于预期，但增速居全省或苏北前列。在统筹做好改革发展稳定各项工作的同时，我们突出抓好以下几个方面：一是切实加强经济运行调度。定期召开经济形势分析会，主动出击加大招商力度，积极搭建招商综合服务平台，促进经济平稳健康发展，主要经济指标保持在合理区间。二是切实加大项目推进力度。开展企业服务年活动，建立完善领导挂钩、市县联动、分级负责的项目推进机制。组织重点项目集中开工活动，扎实推进500个重点项目，128个新开工重点工业项目完成投资128.6亿元，安排专项资金2862万元支持技术改造项目建设。三是切实扩大对外开放交流。全面启动上合组织物流园建设，推动省政府与哈国铁签署战略合作框架协议。一带一路国际农业合作示范区建设全面推进。新亚欧大陆桥安全走廊国际执法合作论坛永久会址和一带一路国际执法安全培训研究中心落户连云港。首批中亚留学生来连入学。四是切实强化政府自身建设。机关公车改革和办公用房清理整顿基本完成。深入开展三严三实专题教育，机关作风进一步转变。自觉接受人大的工作监督、法律监督和政协的民主监督，办理人大代表建议172件、政协委员提案346件，按期办复率100%、满意率达99%。

回顾十二五的丰富实践，工作扎实有力，成就令人振奋，积累的经验更是弥足珍贵。一是必须着眼全局谋划长远发展，找准发展定位，拓展发展空间。二是必须围绕中心狠抓经济工作，坚定不移推进产业强市、工业立市战略，不断增强综合实力。三是必须扩大开放构筑竞争优势，加快汇聚优质要素，擦亮首批沿海开放城市金字招牌。四是必须突出问题导向狠抓改革创新，着力破解制约瓶颈，激发内生发展动力。五是必须坚持共享发展改善民生，把事关广大群众切身利益的各项实事落实好，努力让改革发展成果更多、更公平、更实在地惠及广大群众。

各位代表，过去五年，我们共同生活的这座城市发生了深刻变化，焕发出新的生机与活力。这些成绩凝聚着全市人民的辛勤汗水，饱含着社会各界的贡献付出。在此，我代表市人民政府，向全市人民，向人大代表、政协委员，向各民主党派、工商联、人民团体和社会各界人士，向驻连部队、武警官兵、公安干警，向为连云港改革发展稳定作出贡献的驻连部省属单位，向所有关心支持连云港发展的海内外朋友，表示衷心的感谢和崇高的敬意！

在肯定成绩的同时，我们也清醒的看到，全市经济社会发展还存在薄弱环节。如，经济总量不大，县域经济薄弱，综合竞争力不强，全面小康建设区域不平衡；工业经济发展不快、项目支撑不足，重大临港产业尚未取得明显突破，现代服务业发展滞后，人才、研发、金融等创新要素比较缺乏；集装箱干线港地位尚未确立，港口带动力不强；城市功能不够完善，建设集聚度不高，海滨特色不够鲜明；社会事业布局不够均衡，发展不够充分；政府职能转变还不到位，投资发展软环境有待进一步优化。对此，我们将进一步增强忧患意识和责任意识，在以后工作中采取更加有力措施，切实加以解决。

十三五时期目标任务

十三五是我市深入对接一带一路战略、全面落实江苏沿海地区发展规划的关键时期，是深化改革、推动发展方式转变的战略机遇期，也是全面建成小康社会的决胜阶段。根据市委十一届十一次全会部署，全市十三五发展的指导思想是：高举中国特色社会主义伟大旗帜，全面贯彻党的十八大和十八届三中、四中、五中全会，以及习近平总书记系列重要讲话特别是视察江苏重要讲话精神，深入贯彻省市委决策部署，坚持四个全面战略布局，贯彻五大发展理念，主动适应、把握和引领新常态，抓住用好一带一路等多重叠加机遇，大力实施以港兴市、产业强市、城乡协调发展、创新驱动发展、绿色发展五大战略，加快港产城融合发展，冲刺全面小康社会建设，奋力开创国际化海港中心城市建设新局面。

今后五年，全市经济社会发展总的目标是全面建成小康社会，让人民群众过上更加美好的生活。具体目标要求是：

综合实力显著增强。地区生产总值、公共财政预算收入保持两位数增长，固定资产投资和工业总产值实现翻番。临港工业、重大项目实现大突破，现代农业、现代服务业发展步伐全面加快。徐圩基本建成石化产业基地、精品钢基地，市开发区建成新型科技园区，高新区建设取得阶段性成果，规划建设高铁、空港和海港商圈。中心城市辐射带动能力大幅提升，县域经济跨上新台阶，镇级经济实力明显增强，全市城镇化率达到68%。创新型城市建设全面推进，科技进步贡献率超过60%。

一带一路建设取得新进展。基本建成中哈物流合作基地，形成上合组织出海基地体系布局。港口30万吨级航道全面完成，成为集装箱干线港、枢纽港和一带一路重要出海口。铁路网基本建成，全面进入高铁时代。连云港成为区域性重要交通枢纽和物流中心，全方位开放格局更加完善，东陇海经济带的支撑作用进一步提升，一带一路交汇点核心区、先导区的作用得到有力发挥。

人民生活水平普遍提高。社会就业更加充分，创业致富蔚然成风，城乡居民收入保持两位数增长。多层次社会保障体系更趋完善，社会保障制度更加公平更可持续。教育质量明显提升，教育资源更加均衡。居民健康主要指标达省定标准。建成全覆盖的养老服务体系。人居环境得到提升。公共安全水平提高，人民群众的安全感持续增强。

生态环境明显改善。生产生活方式绿色低碳水平明显提升，单位地区生产总值资源消耗下降、环境质量改善均达到国家控制要求。生态文明建设推进机制和制度体系健全。山体景观特色得到彰显，滨水生态廊道形成，连云港的天更蓝、山更绿、水更清。

社会文明程度大幅提升。社会主义核心价值观得到自觉践行，精神文化生活更加丰富，爱敬诚善的社会风尚更加浓厚，全社会凝聚起强大正能量。人民群众思想道德、科学文化素质明显提高，全社会法治意识增强。文化事业和文化产业繁荣发展，公共文化服务体系更加完善，对外文化交流不断扩大。

全面深化改革持续推进。扎实推进行政体制改革，切实转变政府职能。市场化改革取得显著成效，市场体系更加完善，政府作用得到更好发挥。治理体系和治理能力现代化实现重大进展，形成系统完备、科学规范、运行高效的体制机制。法治连云港建设稳步推进，法治政府基本建成。人权得到切实保障，产权得到有效保护。

实现十三五目标，我们将采取以下主要举措：

一是坚持产业强市、工业立市，着力增强综合经济实力。以供给侧改革为方向，以扩量提质为目标，深入实施转型升级工程、农业现代化工程，贯彻中国制造2025和互联网+行动计划，促进集中集聚集约发展，加快推进农业现代化，建设沿海新型制造业基地，促进服务业提档升级。

二是坚持新型城镇化、城乡一体化发展，着力展现国际海滨城市新风貌。坚持以人的城镇化为核心，尊重城市发展规律，按照五大统筹要求，加快优化城市空间布局，大力提升城市功能、品质，构建现代城镇体系，全面推进港产城融合发展，打造宜居宜业的海滨城市。

三是坚持融入一带一路、推进海陆双向开放，着力提升国际化水平。打造区域性国际航运中心、面向一带一路的国际物流中心和上合组织出海基地，搭建各类开放合作平台，广泛汇聚优质发展要素，深入开展对外交流合作，开放型经济发展取得新成效。

四是坚持深化改革、创新驱动，着力激发内生发展动力。把深化改革贯穿于经济社会发展各个环节，不断突破制约生产力发展的体制性障碍，最大化释放改革红利。把创新作为引领发展的第一动力，把人才作为引领发展的第一资源，深入实施科技创新工程，全面激发经济社会发展活力。

五是坚持绿色发展、低碳发展，着力推进生态文明建设。优化国土空间开发格局，加大环境污染治理力度，着力改善绿色生态环境，大力发展循环经济，完善生态环境保护制度体系，实施蓝天、清水、绿地工程和美好城乡建设工程，建设美丽港城。

六是坚持以人为本、共享发展，着力改善民生和加强社会建设。把新增财力主要用于民生事业，协调推进劳动就业、社会保障、文化教育等各项民生社会事业，不断推进公共服务均等化，深入实施社会治理创新工程和文化建设工程，努力提高人民群众的获得感和幸福感，大力促进社会文明进步。

2016年主要目标和重点工作

2016年是实施十三五规划的开局之年，做好全年工作意义重大。今年工作总的要求是：全面贯彻中央、省委的决策部署，落实市委十一届十一次全会和全市经济工作会议精神，适应经济发展新常态，坚持稳中求进总基调，统筹抓好稳增长、调结构、惠民生、防风险各项工作，在保持经济运行在合理区间的同时，着力加强结构性改革，加快培育新的发展动能，增强持续增长动力，扎实推进一带一路交汇点核心区先导区建设，为十三五时期实现全面达小康、迈上新台阶的总目标开好局、起好步。

今年主要预期目标是：全市地区生产总值增长10%以上，一般公共预算收入增长10%左右，规模以上工业增加值增长11%以上，规模以上固定资产投资增长20%以上，社会消费品零售总额增长11%左右，外贸进出口总额保持正增长，实际利用外资9亿美元，港口吞吐量、集装箱运量分别增长5%，城乡居民收入与地区生产总值保持同步增长，研发投入占GDP比重达1.7%，居民消费价格指数不高于省控线，节能减排完成省定任务。

围绕实现上述目标要求，着重做好以下八个方面工作：

一、坚持产业强市战略，推动经济持续健康快速发展

按照供给侧改革新思路，实施转型升级工程，对接国家、省制造业发展新战略，推动产业向中高端水平迈进、集中集聚集约发展，不断增强综合经济实力。

加快突破工业经济。继续深化企业服务年活动，降低实体经济运营成本，加大指导帮扶力度，推动企业加快发展，全市净增规模以上企业100家。做大做强优势产业。引导新医药、新材料、新能源和先进装备制造业龙头企业提升核心竞争力，带动上下游产业、关联产业发展，着力形成配套完善的产业链条。借助大型药企研发优势，加快打造新医药公共服务平台，积极发展生物医药、天然医药、创新中药、先进医疗器械等产业。发展国内领先的高性能纤维、碳纤维复合材料产业，提升硅材料、新型电子材料产业水平。加快突破核电装备、海洋工程装备、IGCC装备制造、智能制造等先进装备制造产业。改造提升传统产业。以优化布局、结构调整、技术改造和产业链延伸为重点，推动化工、金属冶炼等传统产业转型升级。确保石化产业基地规划尽快获得国家发改委批复，力争盛虹炼化一体化项目通过核准并开工建设。积极承接钢铁产业转移，加快推进钢铁基地建设。加快实施益海精细化工脂肪胺、连云港碱厂技改搬迁等技改项目，完成规模以上技改投资900亿元。积极推进两化融合，全年新增省级两化融合示范试点企业5家。开展质量提升行动，加强质量、标准和品牌建设。淘汰置换落后产能。强化环保、能耗、技术、安全等标准，持续推进小化工、污染企业整治，加快淘汰落后产能，消化一批存量，严控新上增量。妥善清理僵尸企业。开展僵尸企业排查，积极稳妥抓好兼并重组或依法破产，盘活土地、资金、设备等存量资产，释放生产潜力。同时，推动建筑企业提档升级，着力做大做强建筑产业，建筑业总产值达到780亿元。

加快发展现代服务业。以生产性服务业为重点，着力打造服务业集聚区，加快实施服务业重点项目。大力发展物流产业，建成用好港口物流园区、雅仕保鲜冷链物流基地、天马云仓物流配送中心等物流平台建设，加快提升现代物流发展水平。加快发展现代金融业，全年社会融资规模达到530亿元，新引进3家以上金融机构，推动10家以上企业在新三板及区域性股权交易中心挂牌，推动互联网金融健康发展。着力壮大旅游产业，完善旅游公共服务体系，优化旅游市场环境，积极培育旅游新业态，开发智慧旅游系统，启动花果山南侧大型综合游客服务区建设，创成花果山国家5A级景区，推进海洋极地馆、秦山岛等重大旅游项目开发建设。积极发展文化创意产业，加快推进星海湖文化创意产业园、连云港众创谷、西游记影视动漫城等文化园区建设。提升商贸服务业，推进传统商圈改造，推动商贸服务业转型升级。培育新兴服务业态，积极发展服务外包、研发设计、检验检测、电子商务、健康服务等现代服务业。稳定发展房地产业，加快商品房库存消化，满足群众刚性和改善性住房需求。

全面提升项目承载能力。市开发区建成金融大厦、连城新能源创新中小企业园等配套工程，着力打造产业高端化、功能现代化、环境生态化新型科技园区。国家高新区着眼于服务全市创新发展，加快建设具有区域影响力的大数据中心、公共检测中心、创新创业服务中心等载体平台。徐圩新区完善石化、精品钢两大基地及节能环保科技园基础设施，着力完善配套服务，加快建设一体化、大型化、清洁化产业园区。加快推动海洋经济创新示范园区建设，吸引优质要素向园区加速集聚。各县区开发园区加快完善供水、供热、污水处理等基础设施，为产业项目落户创造良好条件，为做强县域经济夯实基础。高度重视镇级经济发展，本着宜农则农、宜工则工、宜商则商的原则，进一步拓宽思路，倾斜扶持乡镇产业园区建设，为推进城镇化奠定扎实的产业基础。深入开展低效闲置土地清理，盘活存量土地13800亩，其中市区8300亩，为项目落户提供土地保障。加强国家电网和输变电重点工程建设，为经济社会发展提供电力支撑。

二、融入一带一路建设，增创开放发展新优势

牢固树立开放发展理念，坚持扩大开放与做优平台互动并进，深入推进一带一路交汇点核心区先导区建设，着力打造海陆东西双向开放门户。

推进综合交通枢纽建设。加快港口建设，优化提升主体港区功能，开工建设30万吨级航道二期工程、30万吨级原油码头，建成旗台作业区2个液体散货泊位。建成徐圩港区东防波堤、赣榆港区防波堤二期工程。完善集疏运体系，推进连盐铁路上合组织物流园、徐圩港区、赣榆港区支线建设。加快实施灌河航道整治工程，开通至淮安、扬州以及长江沿线主要港口的内河航线。稳定并扩大连新亚连新欧运营规模，开行重庆等城市经连云港至日韩多式联运班列。加快连盐、连淮扬镇、连青铁路建设，开工建设徐连高铁。推进市域公路网建设，204国道市区至灌南段路基贯通，加快推进345、235省道建设。优化机场航线网络，推进新机场建设前期工作。

强化开放载体平台建设。加快国际商务中心建设，着力构筑对外开放公共服务体系。推进中哈物流合作基地二期工程、上合组织国际物流园和大宗商品交易中心、东中西区域合作示范区资源集中采购中心建设，打造重点面向中亚、日韩以及中西部地区的大宗货物集散交易基地。综合保税区获批，赣榆、徐圩港区正式开放，灌河港区实现临时开放。举办第三届连博会。开工建设新亚欧大陆桥安全走廊国际执法合作论坛永久会址和一带一路国际执法安全培训研究中心。积极打造人文交流平台，扩大与一带一路沿线国家、地区的经贸合作。

提升开放型经济水平。积极复制自贸试验区经验，推广投资、贸易、金融和综合监管制度四项改革。完善扩大出口综合政策，拓展转口贸易、离岸贸易、租赁贸易等业务，努力培育出口竞争新优势。推动连云港保税展示交易中心、大陆桥跨境电商产业园、东海水晶城大力发展跨境电子商务。加快企业走出去步伐，培育一批具有国际竞争力的本土跨国企业。完善开发园区考核机制，促进开发园区集中集聚集约发展。扎实做好招商引资工作，坚持一二三产并举、大中小项目齐上、国资外资民资项目同抓、市县区镇联动，创新招商引资方式，加强产业链招商、专题招商和以商招商，确保招商引资取得重大突破。

三、加快海滨城市建设，提升城市综合承载力

贯彻新型城镇化和城乡发展一体化战略，完善现代城镇体系。坚持以人为本，完善功能、提升品质、精细管理，建设宜业宜居家园。

强化基础设施配套。完善城市路网体系，新建云宿路、文苑路等30条城市道路，改造秦东门大街西段、中华路等20条老城区道路，打通西盐河河滨路、凤鸣路等10条断头路，提升城区道路通达能力。更新新能源公交车辆400台，新辟优化调整公交线路15条。完善市政公用设施，加快东港污水处理厂建设，提标改造大浦、墟沟污水处理厂，完成海州水厂深度处理主体工程和蔷薇湖输水泵站工程，建成市餐厨废弃物处理厂，改造维修老旧管网20公里，新建各类管网60公里。实施蔷薇河治理、东门五图河整治、猴嘴闸迁建等工程，完成石化产业基地除涝一期工程建设以及绿园路等城区雨水管网整治，启动实施地下综合管廊10公里先导工程。完善农村生活垃圾收运处置体系，所有镇区垃圾无害化处理率达100%、农村达80%。

加快完善城市功能。深化城市功能分区，实施十大类684项建设项目，年内完成投资502亿元。提升综合服务功能，实施教育、医疗、商务、办公等公共服务项目97项，确保海州高级中学、苍梧小学西校区、科技创业城、万达广场建成投用。提升景观环境，启动西凤凰山、凤凰湖公园建设，完成月牙岛、孔望山和桃花涧等景区改扩建，改造后云台山景观，推进以玉带河为重点的河滨绿化、道路提升工程，启动15平方公里海绵城市示范区建设，新增绿地面积300公顷。提升老城区形象，实施白虎山批发市场、人民西路、德邦化工周边及开发区猴嘴等片区旧城改造，完成房屋征收200万平方米，改造70个老旧小区，实施老城区300条、50公里背街小巷整治工程。

健全长效管理机制。强化规划引领，树立系统思维，科学谋划城市成长坐标，推进多规合一。完成城市总规修编报审，编制城市地下管线、综合管廊、海绵城市示范区等专项规划，修编户外广告设置规划。提高城市管理智慧化水平，建立地下管网信息系统、市区污水管网泵站联网监控管理系统、建筑工地实时监控系统等智慧信息平台。开展城市环境综合整治行动，探索建立禁违控违长效管理机制。深化市容环境秩序扁平化、网格化管理。巩固建筑工地及道路扬尘专项整治行动成果，建立健全道路积尘防治机制，开征城市施工工地扬尘排污费。推进城市供水、供热运营机制改革，探索环卫作业市场化，增强公共产品和公共服务保障能力。落实职能部门和属地管理责任，提升物业管理综合服务水平。

四、深入推进改革创新，激发整体发展活力

加大改革攻坚力度，大力实施科技创新工程，积极推进大众创业、万众创新，加快形成引领经济发展新常态的体制机制。

加快突破重点领域改革。进一步转变政府职能，继续推进行政审批制度改革，探索容缺预审限时补报项目审批制度，实施联合审图多评合一等便企措施，推进户籍服务改革公积金流程优化等便民举措。深化财税体制改革，健全政府全口径预算管理体系。理顺市、区财力分配关系，实施新一轮市区财政管理体制改革。推动金融改革先行先试，争取跨境人民币创新试点政策获批实施。深化国资国企改革，优化调整国有资本布局，深化薪酬制度改革，健全运营监督机制和风险管控体系，完善法人治理结构和现代企业制度。推进农村综合改革，完成农村承包土地和小型水利工程确权登记颁证工作，启动第二批经济强镇行政管理体制改革试点。加快文化体制改革，完善国有文化企业的治理结构和运营机制。推进旅游改革创新先行区建设，完善管理经营体制机制，大力整合旅游资源，组建旅游发展控股集团。继续推进城市公立医院改革，加快分级诊疗制度建设。实施机关事业单位养老保险制度改革。推进环保监测监察执法垂直管理。开展海洋与渔业综合执法体制改革试点。

深入推进科技创新。强化企业创新主体作用，实施科技企业培育十百千工程，推广科技金融服务产品，加快培育一批创新型领军企业和科技型中小企业，高新技术企业总数突破200家，新增省级以上企业研发机构20个。加快创新载体平台建设，建设共性技术研发及公共服务平台10个，推动科研基础设施和大型科研仪器开放。加速科技成果转化，组织实施重大科技创新与成果转化项目50项，开发高新技术产品200项，着力打造具有地方特色的产学研合作品牌。切实加大对国家级高新区支持力度，鼓励高新区整合用好现有资源，大力引进外部创新资源，加快建设全市创新发展高地。实施更加积极的创新人才政策，引进各类高端人才500名以上。推进知识产权试点城市建设，加强专利创造运用，培育知识产权优势企业10家，全市授权专利4000件以上。

鼓励支持大众创业。加快构建众创空间，推广创客空间、创新工场等新型孵化模式，发挥行业领军企业、创业投资机构、社会组织等社会力量的主力军作用，发展众创空间5个以上，新增市级创业示范基地10个、省级创业示范基地1个。加快营造有利于创业的制度环境，深化三证合一、一照一码商事制度改革，支持创业投资发展，引导社会资金和金融资本支持创业。大力培育企业家精神和创客文化，支持社会力量举办各类创业培训活动，形成支持创新、投身创业的生动局面。

五、扎实做好三农工作，推进农业农村现代化

以保障农产品有效供给和促进农民增收为核心，深入实施农业现代化工程，加快农业转方式、调结构、强基础。

大力发展现代农业。开展永久基本农田划定，全面推进粮食高产增效创建，建设万亩示范片90个。深入推进农业结构调整，发展适度规模经营，加快打造现代农业示范带，新增土地流转10万亩、设施农（渔）业7万亩，生猪规模养殖比重达65%。壮大新型农业经营主体，提高家庭农场发展质量，强化农民合作社规范管理，扶持龙头企业做大做强，新增销售超亿元龙头企业5个。加快提升园区主导产业和综合配套，建成全产业链现代农业园区5个。全面落实支农政策，建立农业融资担保体系，健全政银农合作长效机制，涉农贷款增长10%。加快建设连云港国家农业科技园区，完善农业人才、科技支撑体系，加强与江苏农职院等高校科研院所合作，新培育职业农民3万人、科技示范户1万户。

促进农村一二三产业融合发展。加强一带一路农业国际合作示范区建设，加快推进东海中澳肉牛、灌云丹育种猪等50个重点项目，建成市级农业信息大数据中心，农产品出口额继续保持全省前列。扎实开展电子商务进农村，建设电商示范村50个，农业电商交易额达20亿元。开展农业休闲观光旅游示范村创建，建成市级乡村旅游示范点20个，乡村旅游收入达30亿元。强化特色品牌培育，启动创建国家农产品质量安全市，争创国家地理标志保护产品，新增无公害、绿色、有机农产品100个。

提高农业物质装备水平。推进高标准农田建设，实施新一轮农田水利建设年，新增旱涝保收田15.5万亩、高标准农田7.5万亩，疏浚整治县乡河道88条、村庄河塘1592个。提升禽畜、水产养殖标准化水平，新改建标准化示范场20个，完成标准化池塘改造2万亩，建成农业物联网应用示范点10个。推动远洋渔业发展，建成高公岛渔港，新增远洋渔船10艘。提升农机化服务水平，秸秆机械化还田率保持50%以上，水稻机械化种植水平达到80%，新增适度规模经营农机合作社20家，新建储粮仓容8万吨。

提升农村公共服务水平。完善城乡发展一体化规划体制，修订镇村布局规划，切实加强小城镇建设。有序推进农业转移人口市民化，构建落户成本科学分担机制，维护进城农民的土地承包权、宅基地使用权和集体收益分配权。深化美丽乡村建设，全面推广六位一体农村环境长效管护机制，建成示范片区6个，新改建农村卫生户厕3.6万户。新改建农村公路300公里，改造桥梁30座，镇村公交开通率达50%。全面解决剩余24万农村人口饮水问题。

六、加强生态文明建设，打造山青水美好家园

落实绿色发展理念，深入实施生态文明建设工程，加强污染防治和生态保护，把生态环境打造成区域竞争优势。

推动绿色低碳循环发展。完成战略环评报告，着力构建科学合理的产业、生活、生态空间格局。积极开展低碳城市试点，推广应用清洁生产先进适用工艺技术，实施万吨企业低碳行动。建立能效在线监测平台，完善一企一档环境管理制度，积极引导企业发展循环经济项目，实现节能减排。全市完成重点节能改造项目30个，力争节约15万吨标煤，污染排放强度和总量双下降，完成省下达任务。

加强大气和水环境治理。深入开展扬尘污染控制，加强工地监管、道路保洁和渣土车辆管理，实施企业脱硫脱硝和除尘设施提标改造，加快推进城区重污染企业搬迁。开展废气排放企业专项整治，严格查处环境违法行为。强化秸秆禁烧和综合利用，巩固常态化管理机制。开展蔷薇河上游污染综合整治行动，实施大浦河、排淡河等河道清淤疏浚，着力消除城市河道黑臭水体。全面实施水环境区域补偿工作，确保水质断面考核良好。深入推进化工园区专项整治。

加强生态环境建设。实施生态文明建设七大行动，市开发区建成国家生态工业示范园区，海州、连云建成国家级生态区，灌云、灌南建成省级生态县。严厉打击非法开山采石，强化废弃塘口整治复绿。严格落实耕地保护和生态红线制度，开展最严格水资源管理考核。加强临洪河口湿地等生态保护，启动花果山国家森林公园创建，推进国家级海洋牧场示范区和海洋生态文明示范区建设，加快构建核心生态网络体系。新增造林8万亩，林木覆盖率达到29.4%，创成全国绿化模范城市。完成秦山岛生态整治修复示范项目，建设海洋环境在线监测系统，构建海洋生态安全保障体系。

七、持续推动民生改善，提高人民群众幸福感

深入实施民生幸福工程，以十项重点民生工程48件实事为抓手，加大民生投入力度，增加公共服务供给，让老百姓的幸福更有质感。

拓宽居民收入渠道。把解决百姓就业作为民生改善的首要任务，作为脱贫致富、居民增收的重要举措。实施更加积极的就业创业扶持政策，鼓励支持返乡创业带动就业，新培育私营企业1万户、个体工商户2万户，新增城镇就业5万人、转移农村劳动力2.5万人。实施大学生就业促进计划，引领大学生创业1000人。加大对劳动密集型企业、中小微企业扶持力度，创造更多就业岗位。推进企业建立和完善工资集体协商制度，稳步提高城镇职工工资性收入。探索建立村级经济增收长效机制，拓宽农民持续增收渠道。启动实施新一轮脱贫致富奔小康工程，以基础设施建设为突破口，加大集中连片贫困地区帮扶力度，实现40个经济薄弱村、12万低收入人口更高标准脱贫。

提高社会保障水平。实施全民参保计划，城镇职工五险平均新增参保人员1.2万人以上，城乡居民参保率进一步提高，新增被征地农民100%刚性进保。完善城乡医疗保险政策，稳定住院费用报销比例。完善养老服务体系，推进医养融合，养老床位总数达到每千名老人35张。推进保障性安居工程建设，建成公租房1400套。统筹城乡低保、医疗救助、应急救助、慈善救助等政策，形成政策衔接、制度整合、机制完善的救助体系，保障困难群众基本生活。加强城市流浪乞讨人员救助管理，实施农村留守儿童关爱工程。

加快发展社会事业。以办人民满意教育为目标，推进学前教育优质普惠发展、义务教育资源均衡化发展、高中教育特色多样发展。改造中小学校舍30万平方米，新建幼儿园18所，海州主城区新改扩建中小学校5所。改善提升县、乡镇办学条件，农村学校国家信息化达标率提高到80%。强化教师队伍尤其是农村教师队伍建设，培育农村骨干教师550名。完善现代职业教育体系，推动职业教育特色发展、打造品牌。推进现代医疗卫生体系建设，提升县、乡、村三级医疗卫生机构建设水平，新增省级示范卫生院（中心）6个、卫生室（站）14个，赣榆区人民医院加快创成三级乙等医院。建成市一院新海新区医院、市妇幼保健院新院区门诊楼，开工建设市康复医院新院区。依法实施全面两孩政策。着力打造健康连云港，加大健康教育宣传力度，提升健康医疗水平，积极发展健康产业，推进健身设施建设，着力培育健康活动社会性组织，推动群众广泛开展文体活动，为增进全市人民身心健康创造条件。深入实施文化建设工程，大力推进文艺精品创作，加强公共文化服务标准化建设，文化艺术城基本建成，档案馆新馆投用。推进省公共体育服务体系示范区创建，加快全民健身中心建设，提升体育设施运营管理水平。加强全媒体城市综合信息平台建设，完善APP社会服务功能，满足市民多样化的信息需求。

实施社会治理创新工程。深化群众性精神文明创建。推广全民阅读活动，实施全民科学素质提升行动。推进多层次多领域依法治理，拓展和规范法律服务，启动七五普法，提高社会依法自治水平。深入推进三社联动，乡镇（街道）政社互动运行机制覆盖率达90%，县区、乡镇（街道）社会组织孵化基地建设率分别达100%、20%。加强宏观质量管理和食品药品市场监管，进一步强化安全生产工作，优化应急联动和防灾减灾救灾机制。健全完善价格调控体系，保持市场价格平稳运行。深化平安港城建设，加强现代信息科技运用，强化基层基础建设，创新完善立体化社会治安防控体系。畅通信访渠道，加强信访矛盾源头防范，推动信访形势进一步好转。提升政府热线服务水平，及时回应群众诉求。加强国防动员、双拥共建和民兵预备役工作，推动民族宗教、气象、地震、地方志、红十字、见义勇为和关心下一代等事业再上新台阶。

八、加强政府自身建设，不断提升政府履职能力

形势的发展、事业的开拓、人民的期待，要求我们加快建设服务型、效率型、务实型、法治型、廉洁型政府，增强政府执行力和公信力，促进治理体系和治理能力现代化。

建设服务型政府。服务人民、服务发展是人民政府的根本宗旨。坚持把为民造福作为第一准则，推动更多公共资源向民生倾斜，建立更加完善的公共服务体系，努力满足人民群众对美好生活的向往。坚持把尊重民意作为第一导向，深入基层、企业、群众，准确把握基层的呼声、企业的需求、群众的期盼，切实做到问政于民、问需于民、问计于民。坚持把群众满意作为第一标准，畅通政府与公众互动渠道，让政府工作更多地由群众来评判。

建设效率型政府。提升效率是加快发展的内在要求。坚持简政放权、放管结合、优化服务，深入推进行政审批制度改革和政府职能转变，该放的权放到位，该管的事务管住管好，不断释放市场活力和社会创造力。坚持管理创新和制度创新，着力发挥市、区（县）两级积极性，加快形成权责一致、分工合理、决策科学、执行顺畅、监督有力的行政管理体制。坚持立说立行、马上就办的工作作风，着力提高各级政府部门办事效率，切实解决群众、企业反映的突出问题，为大众创业、万众创新提供良好环境。

建设务实型政府。求真务实是事业兴旺的关键所在。牢固树立思想务实，举措求实，工作落实的理念，竭力抓发展，全力惠民生，量力搞建设。思想务实就是坚持实事求是，既高举加快发展大旗，自我加压提升标杆，又坚决不搞形式主义，不做表面文章，不搞虚假增长，不搞劳民伤财的形象工程。举措求实就是保持战略定力，狠抓项目建设，扩大有效投入，坚定不移推进产业立市战略，着力解决难事，大力兴办实事，全力以赴推进民生改善。工作落实就是要埋头苦干、真抓实干、敢抓敢干、多谋善干，把确定的各项目标任务不折不扣落到实处。

建设法治型政府。建设法治政府是依法治市的核心内容。坚持依宪施政，依法行政，把政府工作全面纳入法治轨道。积极做好政府立法工作，依法履行职责，努力做到所有行政行为都于法有据，任何政府部门都不得法外设权。坚持依法科学民主决策，完善政府法律顾问制度，开展重大决策举措第三方评估。严格规范公正文明执法，加快推进综合执法，全面落实行政执法责任制。推进政务公开，加快政府诚信建设，定下的民生实事说到做到，对企业的承诺严格兑现，切实做到言必行、行必果，取信于民，取信于企业，取信于社会，以政府诚信施政引领全社会诚信建设。强化法治教育，弘扬法治精神，让公平、正义的法治价值观成为全社会的共同信仰。自觉接受人大及其常委会工作监督、法律监督和人民政协民主监督，高度重视社会和舆论监督。

建设廉洁型政府。廉洁是政府公信力的基石。自觉践行三严三实，扎实推进惩治和预防腐败体系建设，严格政治纪律和政治规矩，坚持不懈纠正四风，认真落实党风廉政建设责任制，强化一岗双责，切实遵守中央八项规定精神和国务院约法三章，严格规范三公经费等一般性支出。加强政府内部权力制约，坚持用制度管权、管事、管人，深入开展审计监督、行政监察，从体制机制上铲除滋生腐败的土壤。深入推进反腐败斗争，严肃查处各类违法违纪案件，着力解决损害群众利益的突出问题。切实加强党风廉政教育，把廉洁自律准则的各项要求刻印在各级干部心头上、落实到行动中，努力实现干部清正、政府清廉、政治清明。

各位代表！面对全市人民的新期待，我们深感责任重大；站在历史发展的新起点，我们豪情满怀。让我们在市委的正确领导下，倍加珍惜不可多得的历史机遇，倍加珍惜来之不易的发展势头，围绕全年任务以及十三五确定的各项目标，万众一心，奋力拼搏，为建设经济强、百姓富、环境美、社会文明程度高的新港城而努力奋斗！
